# Unilateral right thyroid swelling with contralateral left mediastinal mass of benign ectopic thyroid tissue: a rare case report

**DOI:** 10.1097/MS9.0000000000002993

**Published:** 2025-04-04

**Authors:** Waseem Sajjad, Hassan Bin Aziz, Pakeezah Tabasum, Syeda Maryam Alam, Serwan Mufti Muttayab, Javed Iqbal

**Affiliations:** a King Edward Medical University, Mayo Hospital, Lahore, Pakistan; b Department of Surgery, King Edward Medical University, Mayo Hospital, Lahore, Pakistan; c Department of Surgery, Peoples University of Medicine and Health Sciences for Women, Nawabshah, Pakistan; d Department of Surgery, Liaqat National Medical College, Karachi, Pakistan; e Nursing Department Communicable Disease Center, Hamad Medical Corporation, Doha, Qatar

**Keywords:** ectopic thyroid, mediastinal mass, thoracic surgery, thyroid surgery

## Abstract

**Introduction and importance:**

Ectopic thyroid is a very rare developmental anomaly with difficult diagnostic challenges, especially when located away from its normal anatomical pathway of development and descent. The coexistence of a unilateral thyroid enlargement with a mass in the thoracic region is very rare and can resemble malignancies and tumors complicating the process of diagnosis and management.

**Case presentation:**

A 44-year-old female presented with a unilateral right thyroid swelling, with further investigation revealing a left mediastinal mass. Initial imaging and biopsy raised concerns about possible malignancy. However, a surgical biopsy confirmed benign ectopic thyroid tissue. The patient underwent total thyroidectomy and excision of the mediastinal mass, recovering without complications.

**Clinical discussion:**

This case reports a rare coexistence of thyroid swelling and ectopic thyroid in the mediastinum. An extensive diagnostic protocol is mandatory to exclude any potential malignancies. Careful surgical management is required for optimal outcomes and the long-term well-being of the patient.

**Conclusion:**

This case report emphasizes the significance of considering ectopic thyroid as a differential diagnosis of mediastinal masses. This case report presents information that not only helps to prevent misdiagnosis but also guide on the suitable treatment and management.

## Introduction

Ectopic thyroid tissue (ETT) is a rare developmental disorder specified by the existence of thyroid tissue other than its normal anatomical location in the neck. It can be found anywhere along the way of thyroid descent. Although ETT is most commonly found in the base of the tongue, with lingual thyroid accounting for 90% of cases, it can rarely be found in the mediastinum, heart, esophagus, and duodenum^[1,2]^. Ectopic thyroid gland occurs in one case of every 100 000–300 000 individuals, but it occurs in one of 4000–8000 patients with thyroid disease^[3]^. The unusual coexistence of unilateral thyroid tissue with contralateral mediastinal mass offers a diagnostic dilemma that requires a comprehensive differential diagnosis to rule out other possible etiologies such as metastatic disease, lymphoma, or other mediastinal tumors^[4]^.

**HIGHLIGHTS**

- This paper reports a rare case of a unilateral right thyroid mass accompanied by a contralateral left mediastinal mass.
- It is important to consider mass of ectopic thyroid tissue in the differential diagnosis of mediastinal masses.
- An extensive diagnostic protocol is important while dealing with mediastinal masses to rule out any malignancy or metastasis.
- Post-operative care and follow-up is mandatory in case of total thyroidectomy to prevent any post-operative event and thyroid insufficiency.
- This paper provides extensive insights of co-existence of thyroid swelling with mediastinal mass, its diagnostic protocol, treatment and postoperative care.

Understanding the embryological, anatomical, and clinical aspects of ETT is crucial. Patients suspected that ETT should have a detailed clinical history, radiological investigations including ultrasonography, computerized tomography scan, and if needed fine needle aspiration cytology of tissue for accurate diagnosis and management and to avoid unnecessary interventions^[5,6]^. However, ETT can present with symptoms of compression such as dysphagia, dyspnea, or chest pain, depending on the size and location of mass^[7]^. ETT should be taken into account while making differential diagnoses for mediastinal masses. Imaging test and biopsy can be used to rule out the other relevant diagnoses. The advisable treatment for such cases is surgical resection continent of the size, symptoms, and risk of complications^[8]^. Patients with euthyroidism who are asymptomatic and do not require intervention, and should be followed up for any clinical symptoms^[9]^.

The instance of reporting this case as a rare presentation lies in the substantial diagnostic and therapeutic it presents. This case report has been written according to SCARE guidelines^[10]^.

## Case presentation

A 44-year-old married woman presented to the OPD with complaints of right-sided neck swelling for the past six months and left-sided chest pain for the past two months.

On detailed history, it was found that she was in her usual state of health six months back when she developed right-side neck swelling, initially observed by the patient herself. Swelling gradually increased in size, associated with palpitations, heat intolerance, and increased appetite. Patient also complains of left-sided chest pain for 2 months, dull in character, mild in intensity, partially relieved with medication, not radiating to arm or neck, not exacerbated on exertion, not relieved on rest. She had no complaint of difficulty in breathing or eating or unintentional weight loss. Systemic review was not significant. Past medical history was significant for hypertension. Past surgical history was not significant. Drug history was significant for Neomercazole (methimazole) 5 mg, 2 tabs twice a day, and Inderal (propranolol) 10 mg, 1 tab thrice a day. Her family history, and menstrual history was not significant.

On examination, she was a middle-aged female, well-oriented in time, place, and person at the time of examination. Her vitals were: pulse: 88/min, BP: 140/80 mmHg, RR: 16/min, temp: 98°F (afebrile) and SaO2: 96% at room air. General physical examination was not remarkable. On neck examination, multinodular swelling in the anterior part of the neck, more prominent on the right side was present which was moving with deglutition. Bilateral carotid arteries were palpable. No cervical lymphadenopathy was found. On chest examination, expansion was normal bilaterally with no visible scar marks or growths, and there was equal air entry bilaterally on auscultation. The abdominal, extremities and neurological examinations were all unremarkable.

**Figure 1.**
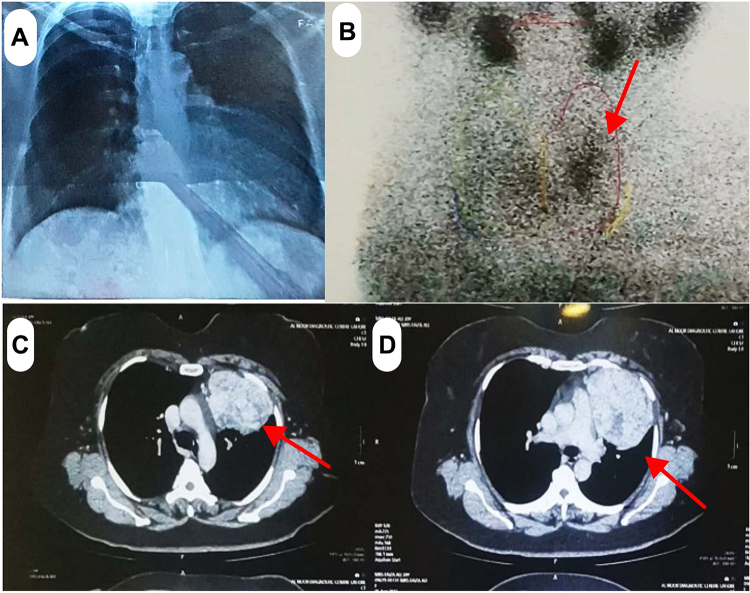
(A) Chest X-ray of the patient. (B) Thyroid scan of the patient. (C) CT chest showing well circumscribed mass. (D) CT chest showing well circumscribed mass.

On investigation, her thyroid function tests were abnormal as shown in Table 1 in comparison to that of post-operative levels. Her serum anti-thyroglobulin level was 60.12 IU (high as compared to normal level which is 4.0 IU). Her serum anti-thyroid peroxidase was 1.0 (normal). An ultrasound of the neck reveals a multinodular goiter with a dominant right nodule. BTA (British Thyroid Association) classification U-2 was established for thyroid nodules. BTA classification is shown in Table 2 for the reference. On chest X-ray (Fig. 1a), the left cardiac border was obscured by a radiopacity in the anterior mediastinum pushing the left hilum posteriorly having a lobulated outline with no intra lesional calcification. Thyroid scan uptake (Fig. 1b) was not that remarkable which could be on account of subacute thyroiditis or reduced thyroid function and/or concurrent use of thyroid-related medication. On the CT chest (Fig. 1c and d), a well-circumscribed heterogeneous mass was noted involving the left upper and mid thoracic region. It measured 106 × 73 x 119 mm. Medially, it was inseparable from the pericardium. Anterolaterally, it was extending up to the chest wall. An ultrasound-guided core (tru cut) biopsy of the chest mass reported the chest mass to be containing of benign thyroid follicles most likely representing ETT. Papillary thyroid carcinoma-like nuclear features were not appreciated. No other teratomatous component was identified.

**Table 1:** Preoperative and post-operative thyroid function tests

Timeline	TSH (0.27-4.2)	Free T3 (3.1-6.8)	Free T4 (12-22)
Initially at presentation (pre-op)	**Figure:** 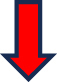 <0.005	**Figure:** 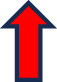 8.40	**Figure:** 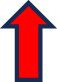 31.57
71 days after surgery (post-op)	0.3	**Figure:** 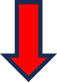 1.8	19.4

**Table 2:** British Thyroid Association (BTA) classification of goiter

Classification	State
U-1	Normal
U-2	Benign
U-3	Intermediate
U-4	Suspicious
U-5	Malignant

The final diagnosis made on the basis of history, examination, and detailed investigations as mentioned above was “benign ectopic thyroid mass in left mediastinum along with right thyroid mass.”

Owing to the patient’s chief complaints (as mentioned in the history), total thyroidectomy and excision of the mediastinal mass were planned. The pre-operative protocol was initiated after finalizing the diagnosis. Total thyroidectomy and Chest mass excision (via thoracotomy) were planned. The patient was prepared for an elective list after clearance from the cardiology and pulmonology department.

**Figure 2.**
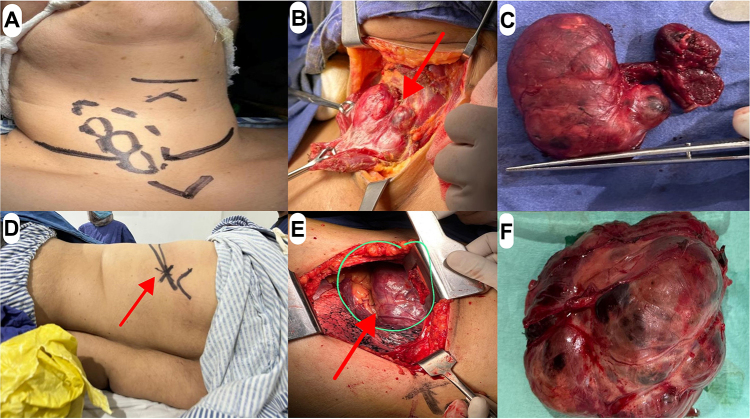
(A) Pre-op image of neck with marking for thyroid surgery. (B) Thyroid exposure through strap muscles. (C) Thyroid gland (removed). (D) Pre-op image of left lateral thorax with marking for thoracotomy. (E) Intra-op image of mediastinal mass exposed and accessed. (F) Image of mediastinal mass (removed).

Intraoperative procedures involved were total thyroidectomy, left posterolateral thoracotomy (as it most commonly adopted approach for removal of mediastinal masses on the surgical floor) with excision of mass, and chest tube placement. Intraoperative findings included: multinodular goiter with right lobe dominant nodule; ETT in the left chest about 9 × 10 cm, which is highly vascular; mass firmly adherent to pericardium; left phrenic nerve running along with undersurface of the mass. Total thyroidectomy was done before the excision of the mediastinal mass. The procedures involved thyroid markings (Fig. 2a) and exposure through strap muscles (Fig. 2b). The total thyroid gland (Fig. 2c) was removed without any remarkable event. The total thyroidectomy was followed by mediastinal mass excision via left posterolateral thoracotomy. The surgery site was marked (Fig. 2d) and dissection was done in the left fifth intercostal space and the fifth rib was exposed and removed for better visualization and accessibility. A clean and neat way was established to mediastinum. After that, the mediastinal mass was accessed and exposed (Fig. 2e). Meticulous dissection was performed to separate the mass from the pericardium while preserving its integrity. The left phrenic nerve was carefully identified and protected throughout the procedure to prevent any injury. These measures ensured the safe removal of the mass without compromising adjacent vital structures. After that, careful removal of the complete mass was done (Fig. 2f). A chest tube was placed and the chest was closed.

Post-operative recovery was uneventful. The patient was allowed for oral intake after 6 hours. Incentive spirometry and chest physiotherapy started. The neck drain was removed on 2nd post-operative day. The chest tube was removed on 5th post-operative day. Patient discharged on the 6th post-operative day.

Adequate follow-up was ensured. It involved: calling the patient on the 10th post-operative day for stitch removal; Tab Thyroxine 50 microgram once daily was started on the 20th post-operative day; the patient was called for follow-up with TSH levels after 3 weeks. No remarkable event occurred and the patient has recovered completely to a quality and standardized lifestyle.

### Discussion

This case report highlights a rare congenital disorder with presentation of unilateral ectopic tissue and contralateral mediastinal mass in a 44-years-old lady. This rare coexistence of thyroid tissue with multinodular goiter in the mediastinum poses significant challenges and management. ETT in the mediastinum presents broad differential diagnoses including primary mediastinal tumors, metastatic disease, lymphoma, and other conditions that mimic this presentation^[11]^. The rarity of ETT in the thoracic cavity lies in its atypical location and asymptomatic nature. It emphasizes the importance of comprehensive diagnostic evaluation in cases where imaging and biopsy results are often inconclusive^[5]^. In short, this case posed various challenges in terms of the coexistence of two similar lesions at different parts of the body without any direct anatomical connection which made the diagnosis challenging. Moreover, the simultaneous management of both lesions was also a challenging part of the surgical floor.

Since cases like this are rare in the available literature especially the management part, which makes this case report valuable in understanding and managing conditions like this. Ultimately, the documentation of such rare cases contributes to knowledge that informs evidence-based clinical practice, particularly in managing rare and complex thyroid conditions. ETT is a congenital anomaly resulting from the failure of the thyroid gland to descend from the primitive foregut to the pre-tracheal region during embryogenesis^[1]^.

The most common region of ETT is the lingual area. However, the occurrence of ETT in the mediastinum is very rare and accounts for less than 1% of all mediastinal tumors^[1,12]^. ETT in the mediastinum can be associated with normal thyroid function and normal thyroid location and should be considered while evaluating mediastinal masses^[13]^. In this case, the clinical signs and symptoms were primarily related to thyroid swelling and mediastinal mass. The right-sided neck swelling associated with hyperthyroid symptoms such as palpitations, heat intolerance, and increased appetite, were suggestive of a functional thyroid nodule. However, the presence of left-sided chest pain, which was dull, non-radiating, and not exacerbated by exertion, initially led to consider a possible cardiac or pulmonary etiology. The prompt further investigation including chest imaging to rule out the underlying causes, revealed mediastinal mass. The subsequent ultrasound-guided core biopsy of chest mass was important in establishing the diagnosis of benign ETT, ruling out malignancy and guiding the surgical approach^[14]^.

This case report requires a surgical approach including total thyroidectomy and excision of the mediastinal mass through thoracotomy. The decision to total thyroidectomy was influenced by the presence of multinodular goiter with a dominant right lobe nodule and to prevent future thyroid-related complications. The excision of mediastinal mass offers additional challenges due to its position and proximity to vital structures such as the pericardium and phrenic nerve^[15,16]^. The initiation of thyroxine replacement therapy was essential in managing the hypothyroid condition of patients following total thyroidectomy^[9]^.

### Future recommendations

Managing thyroid swelling with mediastinal ETT requires a detailed preoperative plan, including imaging to assess vascularity, blood preparation, and hemostatic agents to minimize bleeding, with preoperative embolization considered for highly vascular cases. Surgical challenges, such as prolonged operative time due to adhesions or proximity to vital structures like the phrenic nerve or pericardium, necessitate meticulous dissection and intraoperative nerve monitoring. A multidisciplinary team of thoracic surgeons, endocrinologists, and anesthesiologists ensures comprehensive care. If complete excision is not feasible, alternatives like partial excision or ablation should balance risks and safety, with surgical priority determined by imaging findings and patient stability.

### Conclusion

This case report besides contributing to the limited literature on mediastinal ectopic thyroid, highlights the significance of considering ETT in the list of differential diagnosis while evaluating the mediastinal mass and the associated diagnostic and therapeutic challenges. It also highlights the significance of imaging test and biopsy to accurately diagnose and manage such rare occurrences.

The optimal and successful management of this case involving extensive diagnostic protocol, precise surgical intervention and heedful post-operative measures, serves as an important reference for surgeons and clinicians encountering similar complex cases.

## Data Availability

None.
